# Cupping (Hijama) in Rheumatic Diseases: The Evidence

**DOI:** 10.31138/mjr.32.4.316

**Published:** 2021-12-27

**Authors:** Georges El Hasbani, Ali Jawad, Imad Uthman

**Affiliations:** 1 Division of Rheumatology, Department of Internal Medicine, American University of Beirut Medical Center, Beirut, Lebanon,; 2 Department of Rheumatology, Royal London Hospital, Bancroft Road, London, United Kingdom

**Keywords:** cupping, alternative medicine, safety, rheumatology

## Abstract

Although practiced initially for the alleviation of pain, cupping therapy (Hijama in Arabic) has been used for a variety of medical conditions, including autoimmune and autoinflammatory diseases, with variable outcomes. In recent years, scientific research on the effectiveness of cupping in the treatment of various diseases has accelerated. Relevant literature to identify the types of cupping along with its association with certain rheumatic conditions was screened through a search of the online databases (MEDLINE, PubMed, and Google Scholar) for an indefinite period. Many reports have drawn serious rheumatic side effects which led medical providers to raise the voice against its practice. Moreover, the rare induction of rheumatic conditions has been partly referred to the immunomodulatory effect that cupping exerts in the body. Cupping therapy still needs more evidence to be labelled as therapeutic procedure for rheumatic conditions. Many studies agree that cupping works best when used in combination to pharmacotherapy. Other studies find no clinically significant beneficial role.

## INTRODUCTION

Cupping therapy, Hijama in Arabic, is a traditional alternative medicine that has been practiced in different cultures.^1^ The earliest recorded references to cupping practice are found in the *Ebers Papyrus,* written by Ancient Egyptians in Hieroglyphics about 1550 B.C.^2^ It is noted that cupping use in Egypt dates to 3500 B.C., as documented in hieroglyphic writing.^3^ In addition to ancient Egyptians, the Chinese culture, Arabic medicine, Greek medicine, Prophetic medicine, and the recent European and American medicine, have all practiced the different types of cupping.^3^ Although the therapy has been traditionally performed for the alleviation of pain, numerous medical conditions have been targeted. The American College of Physicians has listed cupping therapy as one of the several methods of complementary medicine for the treatment of chronic lumbar spine pain.^4^ However, cupping therapy is not yet approved for the treatment of any other medical condition.^4^ In recent years, scientific research on the effectiveness of cupping in the treatment of various diseases has accelerated. Autoimmune and connective tissue diseases are among the common diseases that have been treated with cupping, with variable outcomes. Many reports have drawn serious rheumatologic side effects of cupping which led medical providers to raise the voice against its practice. This review briefly describes the types of cupping and its proposed biological mechanism of action before drawing the role of cupping in certain rheumatological conditions. The aim of this review is to shed light on the use of cupping in rheumatology as a beneficial or inadequate method of treatment based on evidence from the literature.

## CUPPING TECHNIQUE

Although several types of cupping exist, such as dry cupping, wet cupping, moving cupping, and fire cupping, the wet and dry types are most commonly used.^5^

Dry cupping involves coating a thick glass cup with alcohol.^6^ A flame is applied and, just before extinguishing it, the coated cup is applied to the patient’s skin. The flame should heat the air in the cup, and not the cup itself.^7^ With the aid of negative pressure, the skin is suctioned into the cup.^8^ The cup is kept in place for around 10 to 15 minutes.^9^

Wet cupping involves bloodletting, and has two main forms. One form is referred to as the cupping, puncturing, and cupping method (CPC), which essentially begins with dry cupping. CPC consists of six steps in total: demarcating the skin, sterilising the area, cupping, puncturing, cupping, and sterilizing once again. The other form is the puncturing and cupping (PC) method, which begins with puncturing. Five steps are instilled here: demarcating the skin, sterilising the area, puncturing, cupping, and sterilising one again. CPC is used more commonly in the Middle East, and is traditionally known as “Al-hijamah”.^10^ The PC method has been commonly used in the Far East and Europe.^10^

## MECHANISM OF ACTION

Cupping has been practiced for four main aims: pain reduction, decrease of inflammation, immunomodulation, and haematological adjustment. For these different aims, many mechanisms of action theories have been proposed.

The three main possible theories that can explain mechanisms of pain reduction are: pain-Gate Theory (PGT), Diffuse Noxious Inhibitory Controls (DNICs), and Reflex Zone Theory (RZT). PGT is one of the most influential theories of pain reduction.^11^ The stimulation of pain receptors increases the frequency of impulses, ultimately leading to closure of the pain gates, and hence, pain reduction.^12^ Subadi and colleagues injected rats taken as a control group with complete Freund’s adjuvant (CFA) at the footpad. Wet cupping therapy was performed at the paralumbar regions 48 hours after the CFA injection in the rats belonging to the treatment group. Pain threshold was assessed 24 hours thereafter. Heat shock protein 70 (HSP70) and ß-endorphin were assessed in the treatment group. The expression of HSP70 and ß-endorphin was significantly higher in the keratinocytes of the treatment group than the control group. Also, the pain threshold after wet cupping therapy was significantly higher in the treatment group than the control group. The authors concluded that wet cupping might be beneficial in pain management through increased HSP70 and ß-endorphin expression.^13^ DNIC describes the inhibitory activity of a wide dynamic range-type of nociceptive spinal neurons triggered by a second, spatially remote, noxious stimulus. Local damage of the skin and capillary vessels induced by cupping may cause a nociceptive stimulus that activates the distraction effects of DNICs.^14^ RZT depends on the hypothesis that signs and symptoms of illness related to one dermatome may be reflected in changes in neighbouring dermatomes.^15^ When the diseased organ sends a signal to the skin through the autonomic nerves, the skin responds by becoming tender and painful with swelling. Skin receptors are activated when cups are applied to the skin. The entire process will result in the increment of the blood circulation and blood supply to the skin and the internal organs through the neural connections.^16^

The anti-inflammatory effect of cupping is mainly mediated by nitric oxide (NO). NO mediates vasodilatation, and regulates blood flow and volume.^17^ Tagil and colleagues implemented wet cupping to 31 healthy volunteers. Venous blood samples and wet cupping blood samples were taken concurrently. Serum NO, malondialdehyde levels, and activity of superoxide dismutase and myeloperoxidase were measured by spectrophotometer. Wet cupping blood had higher activity of myeloperoxidase, lower activity of superoxide dismutase, higher levels of malondialdehyde and NO compared to the venous blood. The authors concluded that wet cupping helps to remove oxidants and decreases oxidative stress.^18^

The immunomodulatory effect of cupping comes through regulating the activity of immunoglobulins and haemoglobin. Numerous studies have assessed the effects of cupping on the immune system. Ahmed et al. showed that cupping significantly reduces the pain and laboratory markers of rheumatoid arthritis activity. Also, it tends to modulate the activity of the innate immune response especially the natural killer cells as well as the adaptive cellular immune through the activity of Soluble Interleukin 2 Receptor SIL-2R.^19^ Mohammad Reza and colleagues evaluated the Interferon Gamma (IFNγ) and Interleukin 4 (IL-4) concentrations in supernatant of vein and cupping blood cultures with or without the presence of phytohemagglutinin (PHA) mitogen. The results showed IFN-γ and IL-4 concentrations in cupping blood samples were higher compared to venous blood samples without presentation of PHA mitogen. In the presence of PHA mitogen, the levels of IFN-γ and IL-4 in cupping blood samples were equally low as in venous blood samples which suggested that lymphocytes in cupping blood samples may not have their natural function, so they cannot properly respond to stimulation of mitogen.^20^ Li et al. reported that cupping can upregulate the oxyhaemoglobin and deoxyhaemoglobin. The red blood cell works to recognize antigens, and eliminate immune complex, tumour cells, and effector cells, as well as bind germs and viruses, and regulate immune function.^21^ Guo and colleagues proposed that physical and biological signals induced by cupping activate the neuroendocrine-immune system, which produces the therapeutic effect.^22^

The haematological adjustment of cupping takes place by detoxification. Several studies reported significant differences in many of the biochemical, haematological, and immunological parameters between the venous blood and the cupping blood.^9^

## METHODS

A PubMed search was conducted with keywords “cupping”, “hijama”, “rheumatology”, “arthritis”, “osteoarthritis”, “fibromyalgia”, “rheumatoid arthritis”, “ankylosing spondylitis”, “gout”, “psoriasis”, “vasculitis”, “scleroderma”, “lupus”, and “myositis” for all available English literature on PubMed Central. All the case reports, case series, cohort studies, and randomised clinical trials reporting the outcomes after the use of any type of cupping for any autoimmune or connective tissue disease were included. Additionally, reference lists of selected manuscripts were checked manually for eligible articles. All articles not indexed on PubMed/MEDLINE were not included. Articles were selected to fit the scope of our topic, reporting the evidence of benefits or inadequacy of cupping therapy in the background of rheumatology.

## RHEUMATOLOGY AND CUPPING

### Fibromyalgia

**Figure 1.**
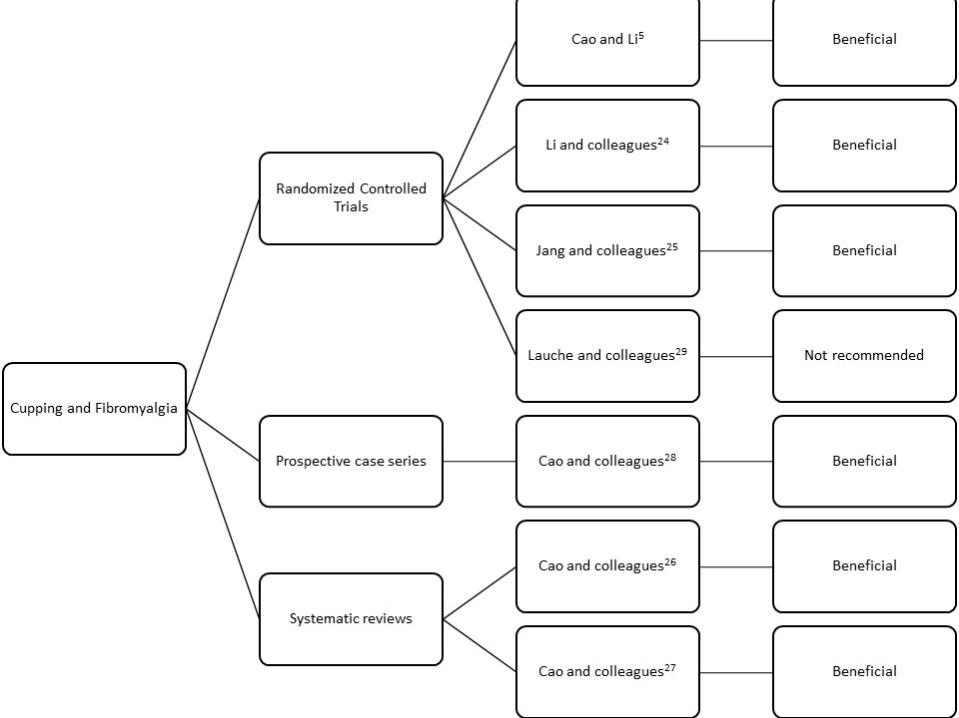
A summary of the randomised controlled trials, prospective case series, and systematic reviews which analysed the effect of cupping in fibromyalgia along with the conclusion of each study.

The fibromyalgia syndrome is characterised by chronic widespread pain in combination with fatigue, cognitive disturbances, sleep disorders, and pronounced somatic and/or psychological distress.^23^practical criteria for clinical diagnosis of fibromyalgia that are suitable for use in primary and specialty care and that do not require a tender point examination, and to provide a severity scale for characteristic fibromyalgia symptoms.\ nMETHODS: We performed a multicenter study of 829 previously diagnosed fibromyalgia patients and controls using physician physical and interview examinations, including a widespread pain index (WPI In total, four early randomised controlled trials (RCTs) showed the efficacy of cupping in fibromyalgia. Two systematic reviews concluded that cupping can be more helpful in treating fibromyalgia when combined with acupuncture and pharmacotherapy than pharmacotherapy alone. One prospective case series found that cupping is beneficial, whereas one recent RCT denied any effect of cupping. **Figure 1** lists all the papers that discussed the effect of cupping in fibromyalgia.

The first Chinese clinical study on cupping as a fibromyalgia treatment was published in 2003. Cao and Li compared the efficacy of acupuncture, cupping, and pharmacotherapy versus pharmacotherapy with regards to pain and depression of fibromyalgia. The authors concluded that the combination therapy alleviated pain and depression better than a single therapy.^5^ Li et al. concluded that a combination of acupuncture with cupping therapy and amitriptyline is significantly more effective than amitriptyline an effective therapy for fibromyalgia syndrome when it came to both pain and depression.^24^ Jang and colleagues used a multi-central randomised controlled method to evaluate the clinical effect of combination of acupuncture, cupping, and pharmacotherapy for treatment of fibromyalgia syndrome. Similar to the previous study, the authors suggested that the combination of acupuncture, cupping, and amitriptyline alleviates pain and depression better than pharmacotherapy alone or complimentary medicine alone.^25^

A meta-analysis by Cao and colleagues showed also that a combination of acupuncture and cupping therapy was better than conventional medications for reducing pain and for improving depression scores in fibromyalgia patients.^26^ Also, another systematic review by Cao and colleagues concluded that Acupoint stimulation therapy, which includes acupuncture and cupping, appears to be effective in treating fibromyalgia compared with medications. However, the authors strongly suggested that further large trials should be performed with better design than the previous work.^27^

A prospective case series by Cao and colleagues was the first to assess the relationship between fibromyalgia and cupping exclusively. Thirty patients diagnosed with fibromyalgia according to the 1990 criteria of the American College of Rheumatology (ACR) were investigated. Pain, assessed via a 10-point visual analogue scale (VAS), and the number of tender points were noted throughout a 2-week treatment with bamboo cup boiled in herbal decoction. A total of 29 patients completed the whole therapy which suggested a reduction in pain and tender points by 48%.^28^

Working according to the 2010 ACR guidelines for fibromyalgia, Lauche et al. aimed to investigate the efficacy of cupping therapy compared to usual care by assessing the pain intensity as well as functional disability, quality of life, fatigue and sleep quality post cupping compared to usual care and sham treatment. Cupping therapy was more effective than usual care to improve pain intensity and quality of life. However, its effects were small and comparable to those of a sham treatment. The authors concluded that cupping could not be recommended for the treatment of fibromyalgia.^29^

### Osteoarthritis

**Figure 2.**
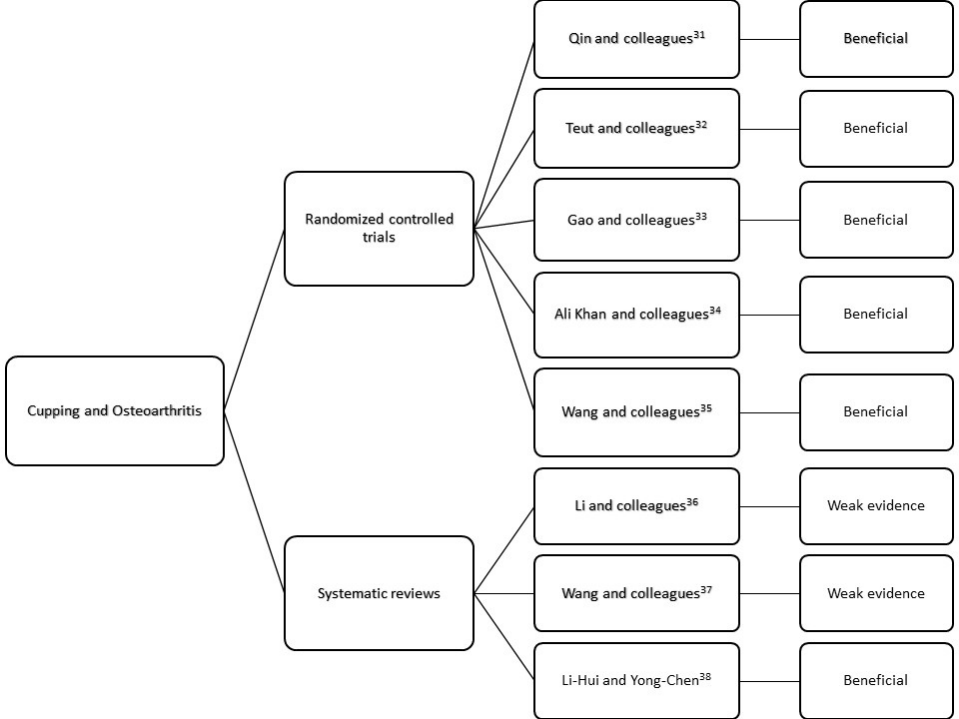
A summary of the randomised controlled trials and systematic reviews which analysed the effect of cupping in osteoarthritis along with the conclusion of each study.

Osteoarthritis (OA) is a clinical syndrome of joint pain which involves localised loss of articular cartilage and new bone formation in places of destructive bone loss at joint margin.^30^”source”:”PubMed”,”event-place”:”London”,”abstract”:”This guideline applies to people with a working diagnosis of osteoarthritis who present for treatment or whose activities of daily living are significantly affected by their osteoarthritis. The management of neck or back pain related to degenerative changes in spine are not part of this guideline. People presenting to health professionals with osteoarthritis complain of joint pain, they do not complain of radiological change. Thus, these guidelines are primarily about the management of older patients presenting for treatment of peripheral joint pain, treatment of the pain itself and of the consequences of such pain for patients who have a working diagnosis of osteoarthritis. The Guideline Development Group (GDG Five RCTs and a single meta-analysis supported the hypothesis that cupping is beneficial for the alleviation of OA pain, whereas three systematic reviews found weak evidence in the support of cupping as a treatment of OA. **Figure 2** lists all the studies which analysed the effect of cupping in osteoarthritis.

In 2008, Qin and colleagues compared the results of treating OA with an integrated Chinese that involves cupping and Western medicine group compared to the Western medicine alone as well as traditional Chinese medicine alone. The randomised controlled study concluded that combined Chinese medicine and Western medicine treatment has rapid and definite therapeutic effect in reducing pain and improving mobility of knee joints and daily living ability in Caucasian patients with knee OA.^31^safety and tolerability of different therapies in Caucasian patients with osteoarthritis (QA Teut and colleagues assessed the effectiveness of dry cupping exclusively in the treatment of knee OA. The pain and quality of life were improved in the treatment group compared to the control group, although the total number of consumed pain killers was not significantly different between both groups.^32^ Gao and colleagues performed a randomised controlled trial to assess the difference in pain between a group of knee OA patients treated only with acupuncture and another group treated with acupuncture plus cupping. The pain score of joint after treatment, as well as the clinical cure rate, was superior in the combination group.^33^50 cases in each one. The comprehensive treatment of fire needles at bones combined with cupping and Tuina on local area of affected knee was applied in the comprehensive group. The Ashi points were mainly selected in the fire needles at bones therapy, once every other day. The cupping and Tuina therapy was adopted once a day. The conventional acupuncture was applied in the acupuncture group, in which Dubi (ST 35 As per the RCT by Ali Khan and colleagues, the efficacy of treatment with cupping therapy in relieving signs and symptoms of knee OA was comparable to that of acetaminophen 650 mg three times per day, in terms of analgesia, anti-inflammatory, and resolution of oedema with minimal and temporary side-effects.^34^ To evaluate the clinical efficacy of the pricking-cupping therapy for knee OA, a multicentre randomised parallel controlled trial was performed by Wang and colleagues. This study concluded that the pain score, stiffness score, physical function score and total score of the Western Ontario and McMaster Universities Osteoarthritis Index (WOMAC) were all reduced after 4 weeks of treatment and during the follow-up visit in the pricking-cupping group and the conventional acupuncture group. However, each score and the total score of WOMAC in the pricking-cupping group were lower than those in the conventional acupuncture group after 4 weeks of treatment and during the follow-up visit.^35^

In contrast to all the positive results, a systematic review performed by Li et al. included 7 RCTs. It concluded that only weak evidence can support the hypothesis that cupping therapy can effectively improve the treatment efficacy and physical function in patients with knee OA.^36^ Also, Wang and colleagues included 5 studies in their systematic review to conclude that there is weak evidence to support the hypothesis that cupping therapy has beneficial effects on reducing the pain intensity and improving the physical function in patients with knee OA.^37^ Recently, Li-Hui and Yong-Chen included 19 eligible studies in their meta-analysis on acupuncture and collaterals cupping therapy for knee OA. The authors found that acupuncture and cupping therapy of traditional Chinese medicine is effective and safe in treating knee OA.^38^

### Rheumatoid Arthritis

Rheumatoid arthritis is an autoimmune disease that has a symmetric, inflammatory peripheral polyarthritis as well as many extra-articular manifestations.

In 2005, Ahmed and colleagues showed that wet cupping significantly reduces the pain and laboratory markers of rheumatoid arthritis (RA) activity. Also, the therapy tends to modulate the activity of the innate immune response mainly the natural killer cells as well as the adaptive cellular immune through the Soluble Interleukin 2 Receptor (SIL-2R).^19^ El Sayed and colleagues showed that Al-hijamah combined with conventional can decrease the rheumatoid factor (RF) levels in RA better than what the conventional therapy can do.^39^ In some patients who suffered from methotrexate-induced leukopenia, Hijama induced leucocytosis, which counteracted the effect of methotrexate.^40^ Cupping has been shown to decrease the levels of serum ferritin which is an inflammatory marker that is elevated in many autoimmune disease including RA.^41^

### Gout

Gout is a broad term for a spectrum of clinical conditions related to an excess of serum uric acid. Gouty arthritis is commonly the first clinical manifestation of gout.^42^ Only one RCT concluded that cupping is beneficial in treating acute gouty arthritis, and one prospective cohort study was able to conclude that cupping is helpful.

Zhao et al. assessed the effectiveness of electro-acupuncture with wet cupping in treating gouty arthritis compared to Probenecid. The wet cupping therapy group had a lower serum uric acid than the Probenecid group.^43^ Zhang and colleagues treated 34 cases of acute gouty arthritis with wet cupping plus herbal medicine. 21 cases were cured and 13 cases improved.^44^

### Ankylosing spondylitis

Ankylosing spondylitis (AS) is a common chronic inflammatory disorder of unknown aetiology, characterised by enthesitis, sacroiliitis, bone hypertrophy, and new bone formation.^45^ One RCT found out that cupping is helpful for the alleviation of AS symptoms, whereas one systematic review found a weak evidence to support that cupping is beneficial for AS.

Wan compared the results of treating a group of patients with AS by acupuncture combined with cupping therapy and treating a control group by simple acupuncture. The author found out that the combination of acupuncture and cupping therapy in the therapeutic effect on AS is better than simple acupuncture, with shorter therapeutic course and lower recurrence rate.^46^ A systematic review by Ma and colleagues which included 5 RCTs concluded that when compared with western medicine alone, cupping therapy plus western medicine has a favourable statistically significant effects on the Bath Ankylosing Spondylitis Functional Index (BASFI) and Bath Ankylosing Disease Activity Index (BASDAI) with low heterogeneity. Furthermore, the effect of the combination of cupping therapy plus western medicine on inflammatory markers is more significant than western medicine alone. However, the authors concluded that when taken together, only weak evidence supported the hypothesis that cupping therapy had potential benefits for patients with AS.^47^

### Psoriasis

Psoriasis is a T-cell mediated autoimmune inflammatory skin disease characterised by skin surface inflammation, epidermal proliferation, hyperkeratosis, angiogenesis, and abnormal keratinisation.^48^ Three case reports in total discussed the topic of cupping therapy in psoriasis. Only one case report found cupping to be beneficial.

Malik and colleagues reported a case of psoriasis with a psoriasis area severity index of 2 who had a decrease in the size of the plaques few days after starting wet cupping. However, some lesions appeared at his elbow after 6 months of treatment.^49^ Yu and colleagues reported a case of Köebner phenomenon induction at the cupping sites in a patient with psoriasis.^50^ Similarly, Vender and Vender reported a case of a biopsy-proven cupping-induced localized psoriasis in a 45-year-old Asian male.^51^

### Vasculitis

The term vasculitis refers to inflammation directed at blood vessels of any size, which leads to destruction of the vessel wall.^52^ One case report supported a beneficence of cupping in treating a form of vasculitis. Another case report illustrated a dermatological side effect of cupping in the use for vasculitis.

Mataix and colleagues reported the case of a 65-year-old male with multiple circular, erythematous, bullous lesions, symmetrically distributed, which occurred after the application of suction cups in the context of polymyalgia rheumatica.^53^ However, Wand reported a case of superficial thrombotic phlebitis treated by wet cupping.^54^

### Scleroderma

The subsets of scleroderma include localised scleroderma, limited cutaneous systemic sclerosis, diffuse cutaneous systemic sclerosis, and systemic sclerosis sine scleroderma.^55^ One case report described an adverse effect of cupping in scleroderma. One case series study found out that cupping can be beneficial for limited scleroderma.

Peter reported a case of a man suffering from arterial occlusion developing deep ulcerations after cupping therapy of circumscribed scleroderma on his right thigh.^56^ Zhou enrolled 52 patients with limited scleroderma and treated them with needling and wet cupping. The study observed that 14 cases obtained clinical short-term recovery, 29 cases had marked effect, and 9 cases had improvement.^57^

No studies have assessed yet the role of cupping in systemic lupus erythematosus (SLE) or in myositis.

## CONCLUSION

As many studies have suggested, cupping therapy (Hijama) still needs more evidence to be labelled as a therapeutic procedure for rheumatological conditions. Many studies agree that cupping works best when used in combination to pharmacotherapy. Still, other studies find no significant beneficial role for cupping. While still rare, the induction of rheumatological conditions by cupping could be explained in part by the immunomodulatory effect that cupping exert in the body. Overall, more studies are still needed to define the role of cupping in rheumatology.
